# A novel digital identification strategy for licorice raw materials: TCM-ID with common/exclusive ion matrices and hierarchical matching

**DOI:** 10.3389/fchem.2026.1918761

**Published:** 2026-10-01

**Authors:** Xianrui Wang, Yu Wu, Lizhi Wan, Jiating Zhang, Fangliang He, Wenguang Jing, Jia Chen, Xianlong Cheng, Yongqiang Lin

**Affiliations:** Institute for Control of Traditional Chinese Medicine and Ethnic Medicine, National Institutes for Food and Drug Control, Beijing, China

**Keywords:** botanical origin, digital identity, *Glycyrrhiza inflata* bat., *Glycyrrhiza uralensis* fisch., *Glycyrrhizae radix* et rhizoma, hierarchical matching strategy

## Abstract

**Introduction:**

*Glycyrrhiza uralensis* Fisch. (GUF) and *Glycyrrhiza inflata* Bat. (GIB) are two official botanical origins of Glycyrrhizae Radix et Rhizoma (GRR). Accurate identification of botanical origin is critical for ensuring efficacy stability and clinical medication safety.

**Methods:**

In this study, an innovative method integrating liquid chromatography-mass spectrometry (LC-MS) with the TCM digital identity (TCM-ID) concept was established for rapid and precise origin authentication of GUF and GIB. Common ion matrix (CIM) and exclusive ion matrix (EIM) of GUF and GIB were systematically constructed to calculate matching confidence (MC) and confidence increment rate (CIR), and a hierarchical matching strategy (HMS) was developed to improve identification accuracy. Meanwhile, the chemometric analysis was adopted to screen differential chemical markers.

**Results:**

The results demonstrated that GUF and GIB could be rapidly and specifically distinguished with thresholds of MC ≥ 70% and CIR ≥ 6.00. Compared to CIM, the EIM exhibited better species specificity, and chemometric analysis identified seven differential markers: Licoricidin, Licoricone, and Glycycoumarin for GUF, and Licochalcone A, Licochalcone B, Echinatin, and Glabrol for GIB.

**Discussion:**

This study provides a novel and feasible approach for botanical origin identification of GUF and GIB, demonstrating that the TCM-ID strategy is a promising method for origin traceability and quality evaluation of GUF and GIB.

## 1 Introduction

*Glycyrrhizae radix* et rhizoma (GRR), commonly known as “Gan Cao”, refers to the dried roots and rhizomes of *Glycyrrhiza uralensis* Fisch. (GUF), *Glycyrrhiza inflata* Bat. (GIB), or *Glycyrrhiza glabra* L. (GGL) (Zhang Y. et al., 2024). It can reinforce the spleen and replenish qi, eliminate phlegm and relieve cough, alleviate acute pain, and harmonize the effects of various TCMs (He et al., 2023; Han et al., 2025). In terms of botanical origin, GUF and GIB are relatively core medicinal sources of GRR, accounting for more than 85% of the GRR market, while GGL is only introduced and cultivated in small quantities in China and is not a mainstream variety (Yang et al., 2024). According to practical conditions, the authentication of GUF and GIB is the top priority in the GRR quality control, which is crucial for ensuring the quality, clinical safety and efficacy of GRR (Qiao et al., 2017).

In order to identify GUF and GIB, numerous scholars have investigated the morphological and microscopic characteristics of GRR (Wang, 2011; Li et al., 2019; Luo et al., 2018; Cao et al., 2009; Yang et al., 2015; National Pharmacopoeia Commission, 2025). On the basis of previous studies, the morphological and microscopic characteristics of these two species are summarized in Table 1.

**TABLE 1:** The microscopic and morphological characteristics for GUF and GIB.

Botanical origin	Microscopic characteristics	Morphological characteristics
*Glycyrrhiza uralensis* Fisch. (GUF)	Cork cells in radial rows, relatively regular; Phloem fiber bundles compact; prism crystals of calcium oxalate present, forming crystal fibers; Reticulate and bordered-pitted vessels; Starch grains abundant; Prismatic crystals of calcium oxalate abundant	Root cylindrical, fairly straight, sparsely branched; Reddish-brown to greyish-brown, exhibiting longitudinal striations; Compact and heavy in texture Cross-section: Strongly fibrous, with few fissures; cambium ring distinct; Odour slight; taste sweet and characteristic
*Glycyrrhiza inflata* Bat. (GIB)	Cork layer relatively thick; Fiber bundles slightly loose; prism crystals of calcium oxalate present, forming crystal fibers; Vessels relatively large, mostly bordered-pitted vessels; Starch grains sparse or indistinct; Prismatic crystals of calcium oxalate large and abundant	Stout, frequently curved, surface rough; Greyish-brown to dark greyish-brown, hard in texture, surface rather rough; Texture rather loose, scarcely floury; Cross-section: Relatively loose with many fissures, moderately floury; Taste sweet, slightly bitter and astringent, with somewhat abundant dregs

Chen et al. explored the differential chemical constituents between GUF and GIB, demonstrating that Licochalcone A and Licochalcone B, Echinatin, and Glabrol serve as characteristic components distinguishing GUF from GIB (Chen et al., 2021). Zhang et al. established fingerprints for GUF and GIB and identified characteristic markers (chalcone A, isoliquiritin, echinatin) using chemometrics, enabling effective discrimination between GUF and GIB (Zhang Y. S. et al., 2024). On the other hand, Kondo et al. realized the identification and differentiation of GUF and GIB based on the rbcL, matK genes and the trnH-trnK1 intergenic region in chloroplast DNA (Kondo et al., 2007). Nishida et al. developed a cost-effective PCR-RFLP method for the rapid identification of GUF (Nishida et al., 2025).

All these studies have contributed to the differentiation of GUF and GIB, yet certain limitations still exist. As shown in Table 1, GUF and GIB have high similarity in their morphological and microscopic characteristics. However, traditional identification relies on the experience of identifiers, which is highly subjective and limited in accuracy, making it easy to cause confusion between the two species. At present, conventional identification methods such as TLC, HPLC, and LC-MS mostly focus on the detection of a small number of chemical components. It can hardly comprehensively reflect the species specificity between GUF and GIB, making it difficult to achieve botanical origin identification (Yang et al., 2017). In addition, molecular biological identification often requires fresh samples, and is cumbersome and time-consuming, which cannot meet the demand for high-throughput and rapid identification of large quantities of TCM (Hou et al., 2023; Jia et al., 2026).

With the advent of the digital and intelligent era of TCM, accordingly, this study innovatively combined LC-MS with the concept of traditional Chinese medicine “digital identity” (TCM-ID) to establish specific “digital identities” for GUF and GIB, and proposes a hierarchical matching strategy (HMS) (He et al., 2025; Zhang et al., 2016; Chen et al., 2024). It aims to develop an accurate, efficient and convenient method for the botanical origin authentication of GUF and GIB, so as to provide scientific support for the quality control, market standardization and rational clinical application of Glycyrrhizae Radix et Rhizoma. Firstly, the GUF and GIB were analyzed through LC-MS and their mass spectrometry were quantified and converted into ion datasets. Secondly, the “digital identity” of GUF and GIB based on common ion matrix (CIM) was obtained from multiple batches of GUF and GIB, respectively. Then the “digital identity” of exclusive ion matrix (EIM) of GUF and GIB were obtained by removing the shared ions. Finally, matching was first carried out using the “digital identity” based on the common ion matrix (CIM), followed by further matching with the “digital identity” derived from the exclusive ion matrix (EIM), yielding matching confidence (MC) and confidence increment rate (CIR). Thus, the rapid authentication of the botanical origin of Glycyrrhizae Radix et Rhizoma was realized using the aforementioned hierarchical matching strategy. Moreover, the differences in the chemical composition of GUF and GIB were also explored using chemometrics.

## 2 Materials and methods

### 2.1 Instrument and raw materials

35 batches of GUF and 35 batches of GIB were acquired by the National Institutes for Food and Drug Control (NIFDC) and botanically identified and verified to meet the 2025 Ch. P requirements by Researcher Cheng Xianlong. All samples were stored in the specimen repository of NIFDC. In addition, the mixed quality control (QC) sample was prepared by mixing GUF and GIB at a ratio of 1:1.10 batches of commercially GRR were purchased for blind sample testing. Supplementary Table S1 presents information on the GRR samples. XPR106DUH electronic balance was purchased from Mettler Toledo Technology (China) Co., Ltd. Waters G2 QTOF high-resolution mass spectrometer. Purified water was provided by Hangzhou Wahaha Group Co., Ltd. Methanol (batch No. EK243-US) and Acetonitrile (batch No. EK823-US) used in this study were supplied by Thermo Honeywell Trading Co., Ltd. Formic acid (batch No. 6624729) was supplied by Dikma Technologies Co., Ltd.

### 2.2 Measurement parameters

The UHPLC-BEH C_18_ column (100 mm × 2.1 mm, 1.7 μm) was applied for chromatographic elution and separation. The column temperature: 35 °C; Flow rate was 0.3 mL/min; Injection volume: 2.0 μL. The gradient elution procedure of Acetonitrile (A) and 0.1% formic acid-water (B) was as follow: 0–0.3 min, 5%A; 0.3–5.5 min, 5%–15%A; 5.5–23.5 min, 15%–50%A; 23.5–40.5 min, 50%–95%A; 40.5–42.0 min, 95%A; 42.0–42.1 min, 95%–5%A; 42.1–45.0 min, 5%A. High resolution mass spectrometry detection was performed under ESI+, ESI- and MS^E^ modes. The mass scanning range was set from 50 to 1200 Da, with a single scan duration of 0.1s. The ion source temperature and desolvation gas temperature were maintained at 120 °C and 400 °C, respectively. The desolvation gas flow and cone gas flow were adjusted to 600 L/h and 50 L/h. The capillary voltage was 3.0 kV in positive ionization mode and 2.5 kV in negative mode, while the collision voltage ranged from 10 to 50 V throughout the measurement.

### 2.3 Sample pretreatment

Each specimen was ground into powder and passed through a 50-mesh sieve. Exactly 0.5 g of each pulverized sample was weighed, then mixed with 20.00 mL of 75% methanol. The mixture was subjected to ultrasonic extraction for 0.50 h under the conditions of 400 W power and 40 kHz frequency. After treatment, the resulting solution was filtered through a 0.22 μm membrane to prepare test solution for subsequent analysis (He et al., 2025).

### 2.4 Method validation

#### 2.4.1 Specificity

To evaluate specificity, mass spectrometric analysis was conducted on 75% methanol (blank), GUF and GIB samples to determine whether the blank solvent interferes significantly with the detection of GUF and GIB.

#### 2.4.2 Accuracy

Using compounds including licochalcone A, leucine enkephalin, and glycycoumarin, the mass error between the calculated and observed m/z values was determined to evaluate mass accuracy.

#### 2.4.3 Precision

The mixed QC sample was analyzed by six consecutive injections. Based on retention time divisions at 10 min, 20 min and 30 min, one quality control point (t_R_-m/z) was selected from each time segment, and a total of four points (Point_A, Point_B, Point_C and Point_D) were used for precision evaluation.

#### 2.4.4 Repeatability

Six replicate mixed QC samples were prepared in parallel and analyzed by mass spectrometry. Based on retention time divisions at 10 min, 20 min, and 30 min, one quality control point (t_R_-m/z) was selected from each time segment, and a total of four points (Point E, Point F, Point G, and Point H) were used for repeatability evaluation.

#### 2.4.5 Stability

The mixed QC sample was stored at a constant temperature of 4 °C and analyzed at 0 h, 4 h, 8 h, 12 h, 24 h and 48 h. Based on retention time divisions at 10 min, 20 min and 30 min, one quality control point (t_R_-m/z) was selected from each time segment, and a total of four points (Point_I, Point_J, Point_K, and Point_L) were used for stability evaluation.

### 2.5 Data preprocessing and analytical workflow

The QI tool was applied for MS information conversion (Zhang et al., 2016). The raw mass spectrograms were converted into a three-dimensional data matrix (t_R_-m/z-I). As shown in Figure 1, after blank elimination, the CIM of GUF (or GIB) can be obtained through taking intersection of ions from different batches of GUF (or GIB) and retain the top 20 ions according to ion intensity. Then, the EIM of GUF (or GIB) were obtained by taking de-intersection and retain the top 20 high-intensity ions. During above data processing, the ions with highly similar retention times (Δt_R_ ≤ 0.20min) and mass-to-charge ratios (Δm/z ≤ 0.1Da) are identified as redundant or interfering ions and subsequently excluded.

**FIGURE 1:**
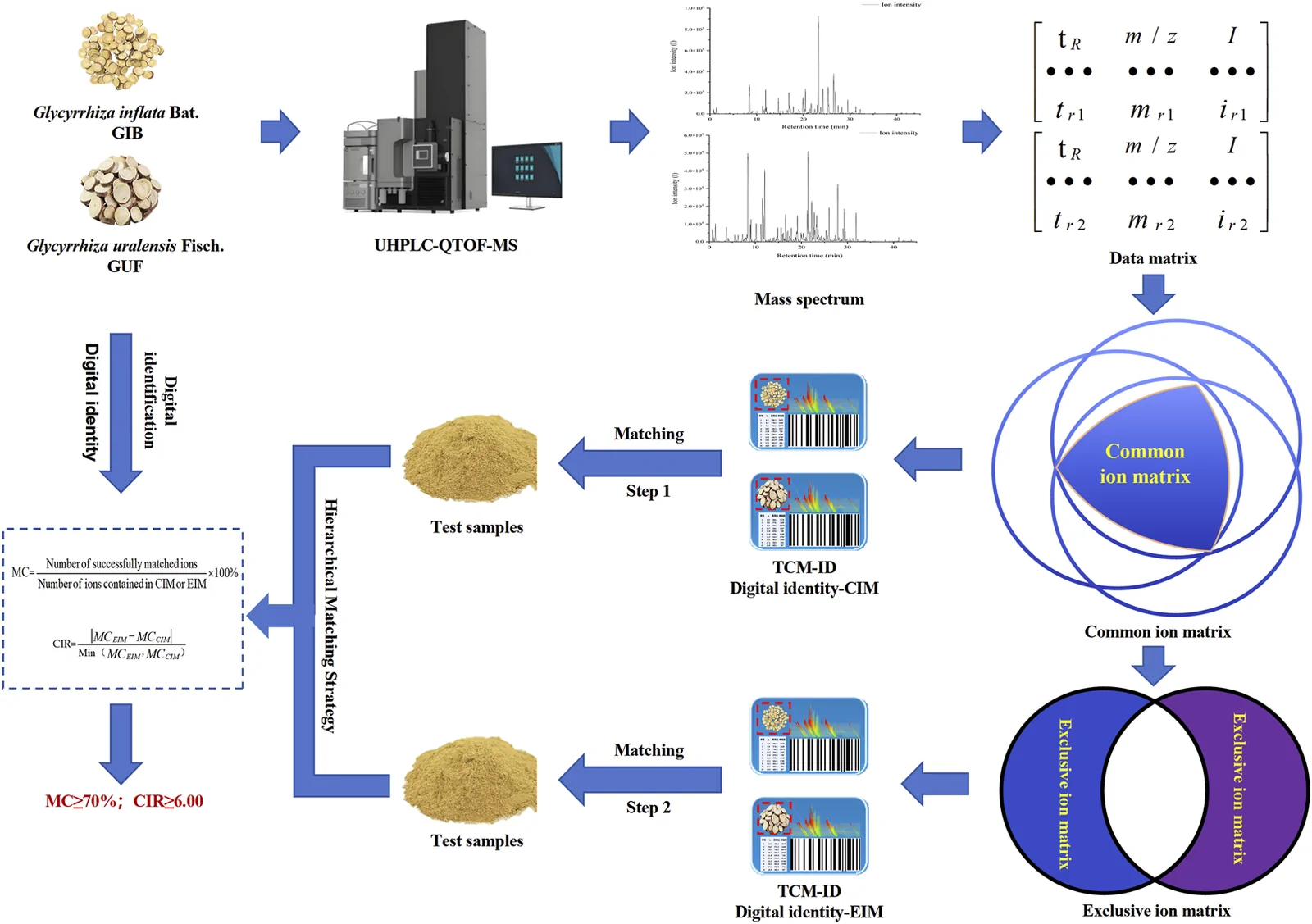
The analytical workflow for identification matching.

Hierarchical matching strategy: first, the matching identification was conducted based on the CIMs of GUF and GIB, followed by further matching with the EIMs of GUF and GIB. Meanwhile, the MC and CIR were calculated and provided for comprehensive evaluation. The MC were calculated using the following equations:

$$\text{MC}=\frac{\text{Number}\;\text{of}\;\text{successfully}\;\text{matched}\;\text{ions}}{\text{Number}\;\text{of}\;\text{ions}\;\text{contained}\;\text{in}\;\text{CIM}\;\text{or}\;\text{EIM}}\times 100\%$$

The successfully matched ions refer to the ions in test samples show highly similar tR and m/z to those in CIM or EIM, satisfying the thresholds of ΔtR ≤ 0.20min and Δm/z ≤ 0.1 Da.

When the same test sample is matched with the CIMs (or EIMs) of GUF and GIB, respectively, the CIR is calculated using the following equations:

$$\text{CIR}=\frac{\left|MC_{GUF}-MC_{GIB}\right|}{\mathrm{Min}\left(MC_{GUF},MC_{GIB}\right)}$$

The *MC*_*GUF*_ represents the matching credibility obtained by matching the test sample with TCM-ID (CIM and EIM) of GUF. The *MC*_*GIB*_ indicates the matching confidence of the same test samples matched with TCM-ID (CIM and EIM) of GIB. The |*MC*_*GUF*_ *-MC*_*GIB*_| refers to the increment of MC obtained by matching the same test samples with TCM-IDs of GUF and GIB. The Min (*MC*_*GUF*_, *MC*_*GIB*_) represents the minimum value of *MC*_*GUF*_ and *MC*_*GIB*_. There is a special circumstance in which Min (*MC*_*GUF*_, *MC*_*GIB*_) is 0%, and the CIR is defined as positive infinity, which indicated that the tested sample cannot be the GUF or GIB corresponding to an MC value of 0%.

## 3 Results and discussion

### 3.1 Method validation results

As shown in Figure 2, the blank solvent (75% methanol) exhibited no significant peaks within 0–40 min and showed minimal interference with the analysis of GUF and GIB, confirming satisfactory method specificity. As shown in Supplementary Table S2, the mass errors of all compounds were within ±5.0 ppm, indicating good mass accuracy. Supplementary Table S3 shows the results of precision evaluation. The RSD values for precision were all below 0.14%, indicating good instrumental precision. Supplementary Tables S4 and S5 show the results of repeatability and stability, respectively. The RSD values were all below 0.70%, which indicated that relatively good repeatability and stability. In conclusion, the results of method validation demonstrate that the method established in this study can meet the requirements for MS analysis of samples.

**FIGURE 2:**
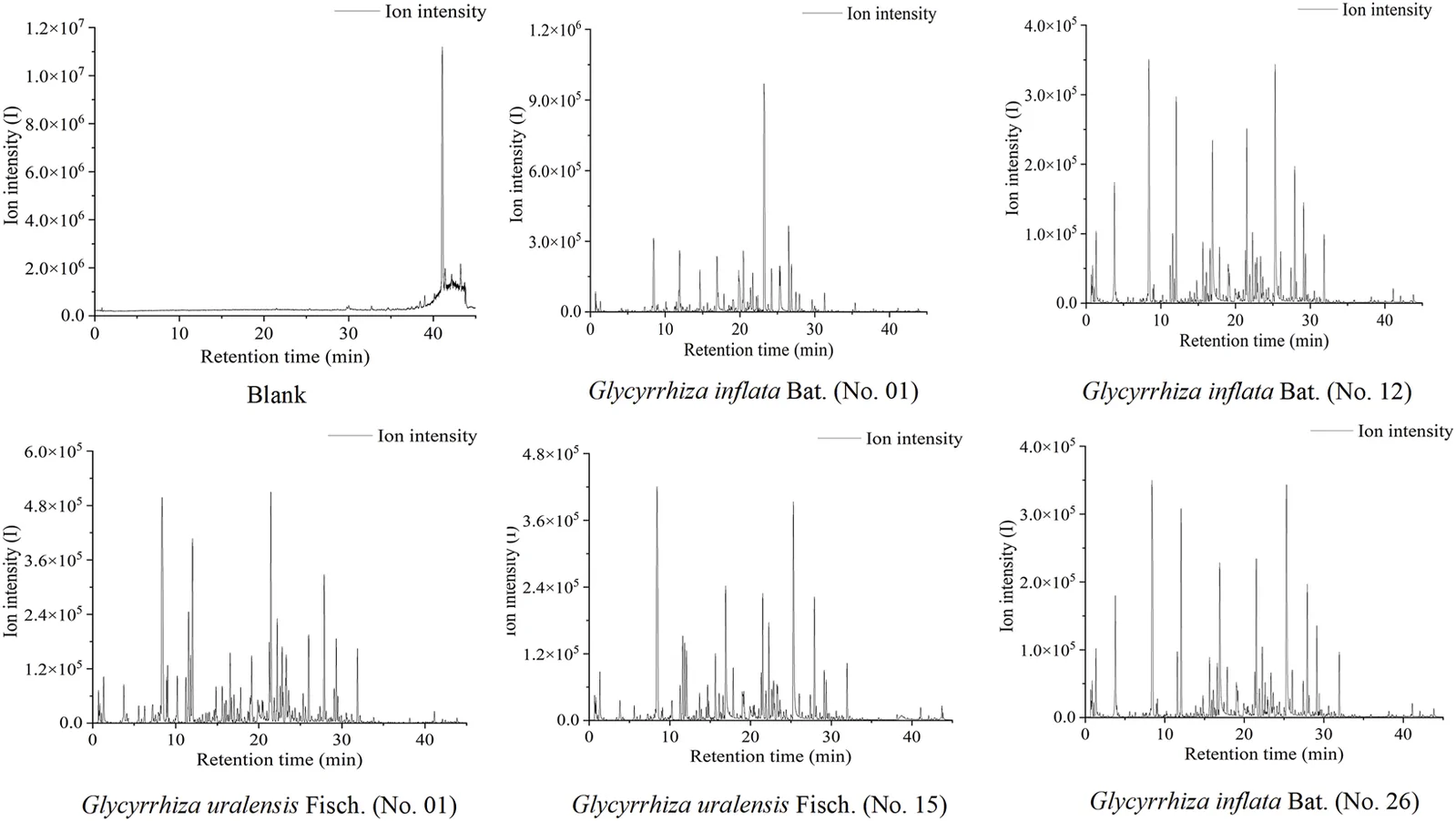
The base peak ion chromatograms of blank, GUF and GIB (Specificity).

### 3.2 Mass spectrometric characterization

Figure 2 shows the results of MS characterization. With 75% methanol as the blank solvent, no obvious matrix effect or interference was observed during the detection of GUF and GIB. In terms of the total ion chromatograms (TICs) of GUF and GIB, they exhibited high similarity, indicating that most of their chemical components overlapped, which posed challenges for the identification of their botanical origins. More specifically, It is relatively difficult to conduct botanical origin identification of GUF and GIB solely based on the comparison of total ion chromatograms (TICs). Therefore, to achieve the authentication of GUF and GIB based on their chemical compositions, novel strategies are required: the concept of TCM-ID is proposed for the identification of traditional Chinese medicines, starting from digital quantitative characterization and ion composition matrix while also taking into account the information of unknown chemical components.

### 3.3 Digital quantitative characterization

To establish the common ion matrix (CIM) and exclusive ion matrix (EIM) of GUF and GIB, data conversion was performed using specialized progenesis software (Chen et al., 2024). The ion numbers of GUF and GIB employed for the construction of CIM and EIM are presented in Table 2.

**TABLE 2:** The ion number of GUF and GIB samples.

Herbs	Lot	Count	Herbs	Lot	Count	Herbs	Lot	Count	Herbs	Lot	Count
GUF	GUF02	9,453	GUF	GUF18	9,153	GIB	GIB01	4,455	GIB	GIB19	6,195
	GUF03	9,336		GUF19	9,110		GIB04	4,232		GIB20	6,220
	GUF04	9,473		GUF21	7,995		GIB05	4,208		GIB21	4,036
	GUF06	9,421		GUF22	8,036		GIB06	4,156		GIB22	4,033
	GUF09	9,426		GUF23	8,061		GIB07	4,174		GIB24	4,091
	GUF10	9,312		GUF24	8,030		GIB09	4,159		GIB25	4,194
	GUF13	9,382		GUF26	7,930		GIB11	6,673		GIB26	4,022
	GUF14	9,312		GUF27	7,717		GIB12	6,496		GIB27	4,066
	GUF15	9,291		GUF28	7,477		GIB16	6,562		GIB28	4,053
	GUF17	9,338		GUF29	7,590		GIB18	6,367		GIB29	6,192

In essence, variations in ion number reflect, to some degree, the differences in their chemical components. As presented in Table 2, the ion quantity of GUF ranged from 7,477 to 9,473 and the ion quantity of GIB varied from 4,022 to 6,673 due to differences in origin, batch, years, storage conditions and other factors, which agreed with practical situations. That is to say, the GRR samples derived from the same botanical origin will inevitably exhibit discrepancies in their chemical profiles, as a result of variations in geographical source, growth years, harvest batches, and other related factors. Conversely, even if the origin, age, or harvest batch differs, as long as the licorice is derived from the same source, it will inevitably contain the same chemical components, regardless of any variations in its chemical compositions. Therefore, comprehensively considering the influences of different production regions, growth years, harvest batches and other factors is crucial for establishing the common ion matrix (CIM) and exclusive ion matrix (EIM) of GUF and GIB (Wang X. R. et al., 2024).

### 3.4 Extraction and identification of common ion matrix

#### 3.4.1 Extraction of common ion matrix

The GUF and GIB samples presented in Table 2 were used to construct their respective CIMs. Ultimately, the ion information (t_R_-m/z-I) that recurred consistently across GUF and GIB samples from different origins, growth years, and multiple batches, with the top 20 ion intensities, was separately screened out to serve as the CIMs for GUF and GIB, respectively. The CIMs of GUF and GIB are shown in Tables 3 and 4.

**TABLE 3:** The common ion matrix (CIM) of *glycyrrhiza uralensis* Fisch. (GUF).

t_R_/min	m/z	I	t_R_/min	m/z	I	t_R_/min	m/z	I
27.91	425.23	4,26,435	17.81	839.41	1,92,708	19.14	369.13	1,75,343
8.36	257.08	3,81,492	29.07	423.18	1,92,545	23.32	383.15	1,55,347
21.47	369.13	3,19,816	25.98	383.15	1,85,143	27.56	707.28	1,51,091
18.97	823.42	2,89,957	11.53	419.13	1,85,109	8.34	837.26	1,48,113
15.62	985.47	2,81,798	21.31	353.10	1,81,084	16.08	881.42	1,46,280
22.23	355.12	2,44,283	31.95	439.25	1,80,813	11.79	431.13	1,40,139
29.35	423.22	2,06,220	12.01	257.08	1,80,160	—	—	—

**TABLE 4:** The common ion matrix (CIM) of *glycyrrhiza inflata* Bat. (GIB).

t_R_/min	m/z	I	t_R_/min	m/z	I	t_R_/min	m/z	I
25.30	270.32	3,62,901	24.20	337.11	1,84,174	20.06	823.42	97,313
19.06	823.42	2,53,450	14.68	271.10	1,69,532	11.60	419.13	89,980
8.40	257.08	2,33,540	3.80	262.19	1,35,440	20.46	353.13	89,375
15.63	985.47	2,29,368	12.08	257.08	1,27,169	8.38	837.26	87,776
22.48	339.16	1,98,867	16.12	881.42	1,26,108	20.35	453.34	81,482
11.93	287.09	1,94,182	19.09	453.34	1,03,498	22.27	355.12	75,037
17.87	839.41	1,88,253	11.31	255.07	1,02,646	—	—	—

#### 3.4.2 Identification of common ion matrix

The CIMs of GUF and GIB were used for matching tests to return matching confidence (MC) and confidence increment rate (CIR). Table 5 shows the MC and CIR results of GUF experimental samples based on CIMs of GUF and GIB.

**TABLE 5:** The MC and CIR results of 10 batches of GUF experimental samples.

Herbs	Batches	Match ions	CIM of GUF or GIB	MC (%)	CIR
GUF	GUF01	19	GUF-20	95.0	0.19
		16	GIB-20	80.0	
	GUF05	20	GUF-20	100.0	0.54
		13	GIB-20	65.0	
	GUF07	18	GUF-20	90.0	0.50
		12	GIB-20	60.0	
	GUF08	20	GUF-20	100.0	0.43
		14	GIB-20	70.0	
	GUF11	18	GUF-20	90.0	0.38
		13	GIB-20	65.0	
	GUF12	19	GUF-20	95.0	0.58
		12	GIB-20	60.0	
	GUF16	20	GUF-20	100.0	0.54
		13	GIB-20	65.0	
	GUF20	20	GUF-20	100.0	0.25
		16	GIB-20	80.0	
	GUF25	18	GUF-20	90.0	0.06
		17	GIB-20	85.0	
	GUF30	19	GUF-20	95.0	0.06
		18	GIB-20	90.0	
	GUF31	20	GUF-20	100.0	0.25
		16	GIB-20	80.0	
	GUF32	20	GUF-20	100.0	0.43
		14	GIB-20	70.0	
	GUF33	20	GUF-20	100.0	0.25
		16	GIB-20	80.0	
	GUF34	20	GUF-20	100.0	0.18
		17	GIB-20	85.0	
	GUF35	18	GUF-20	90.0	0.20
		15	GIB-20	75.0	

As listed in Table 5, all GUF test samples exhibited MC ≥ 90.0% when matched against the CIM of GUF, and all GUF test samples exhibited MC ≥ 60.0% when matched against the CIM of GIB. At the same time, the CIR reflects the degree of variation in matching confidence, ranging from 0.06 to 0.58, indicating that the improvement in matching confidence was not substantial. Overall, the GUF samples consistently exhibited preferential matching to the common ion matrix (CIM) of their own botanical origin, while partial alignment with the CIM of GIB was also observed. In addition, Table 6 shows the MC and CIR results of GIB experimental samples based on CIMs of GUF and GIB.

**TABLE 6:** The MC and CIR results of 10 batches of GIB experimental samples.

Herbs	Lot	Match count	CIM of GUF or GIB	MC (%)	CIR
GIB	GIB02	19	GIB-20	95.0	0.73
		11	GUF-20	55.0	
	GIB03	18	GIB-20	90.0	1.25
		8	GUF-20	40.0	
	GIB08	20	GIB-20	100.0	0.82
		11	GUF-20	55.0	
	GIB10	20	GIB-20	100.0	1.22
		9	GUF-20	45.0	
	GIB13	20	GIB-20	100.0	0.11
		18	GUF-20	90.0	
	GIB14	19	GIB-20	95.0	0.12
		17	GUF-20	85.0	
	GIB15	18	GIB-20	90.0	0.06
		17	GUF-20	85.0	
	GIB17	18	GIB-20	90.0	0.50
		12	GUF-20	60.0	
	GIB23	18	GIB-20	90.0	0.13
		16	GUF-20	80.0	
	GIB30	20	GIB-20	100.0	0.54
		13	GUF-20	65.0	
	GIB31	19	GIB-20	95.0	0.72
		11	GUF-20	55.0	
	GIB32	20	GIB-20	100.0	0.25
		16	GUF-20	80.0	
	GIB33	20	GIB-20	100.0	1.00
		10	GUF-20	50.0	
	GIB34	20	GIB-20	100.0	1.22
		9	GUF-20	45.0	
	GIB35	18	GIB-20	90.0	0.50
		12	GUF-20	60.0	

As listed in Table 6, all GIB test samples reached MC ≥ 90% when matched against the CIM of GIB. Nevertheless, when matched with the CIM of GUF, GIB test samples displayed matching confidence (MC) values ranging from 40% to 90%. The CIR reflects the degree of variation in matching confidence, ranging from 0.06 to 1.25, indicating that the improvement in matching confidence varied considerably among different test samples. Further, the MC values were categorized into two groups for non-parametric testing: Group I comprised the matching confidence of GUF and GIB against their own CIMs, while Group II comprised the matching confidence of GUF and GIB against non-autologous CIMs. As shown in Supplementary Table S6, the *p*-value was less than 0.01, indicating a statistically significant difference and demonstrating that the GUF and GIB test samples retained a distinctly higher matching preference for their own CIM.

Based on Tables 5 and 6, despite the relatively large fluctuation in CIR, both GIB and GUF test samples showed significantly stronger matching affinity toward their respective CIMs, with MC values of no less than 90.0%. It indicated that the CIMs of GUF and GIB facilitate their botanical origin authentication to some degree but they still can not achieve highly accurate and unambiguous differentiation. The above situation can be explained by the fact that GUF and GIB samples are capable of partial matching with non-autologous CIMs, which significantly weakens the significance of identification parameters. Although the CIMs fully considered variations in the geographical origin, production batch and growth year of GUF and GIB, they do not incorporate the interspecific differences between GUF and GIB. Both GUF and GIB belong to GRR and share certain identical and stable chemical constituents. Therefore, as shown in Tables 3 and 4, there are shared ion information in the CIMs of GUF and GIB such as Liquiritigenin (8.36 min_m/z 257.08; 8.40 min_m/z 257.08) and Liquiritin (11.53 min_m/z 419.13; 11.60 min_m/z 419.13). In summary, although identification based solely on the CIM cannot achieve efficient and accurate differentiation between GUF and GIB, it can serve as a preliminary screening step in a hierarchical matching strategy (HMS). This approach requires that test samples exhibit preferential matching to their own CIM, with a matching confidence (MC) threshold of no less than 90%.

### 3.5 Extraction and identification of exclusive ion matrix

#### 3.5.1 Extraction of exclusive ion matrix

Further, the EIMs of GUF and GIM were obtained by removing the shared ions between the two species, and the top 20 ion features ranked by intensity were separately retained as the final EIMs for GUF and GIB, respectively. Tables 7 and 8 show the EIMs of GUF and GIB.

**TABLE 7:** The exclusive ion matrix (EIM) of *glycyrrhiza uralensis* Fisch. (GUF).

t_R_/min	m/z	I	t_R_/min	m/z	I	t_R_/min	m/z	I
25.64	367.12	63,067	25.60	733.23	6,908	18.84	499.31	5,448
21.34	300.06	14,770	20.36	537.33	6,804	17.32	501.32	5,162
41.34	910.75	14,351	23.98	483.31	6,601	17.31	459.24	5,073
21.83	969.65	13,577	24.92	902.51	6,441	41.74	908.74	4,943
14.86	1207.54	10,726	31.19	937.66	6,202	28.85	426.20	4,862
41.48	891.71	91,45	41.39	875.71	5,988	21.72	483.31	4,558
34.00	421.20	77,67	41.68	910.75	5,793	—	—	—

**TABLE 8:** The exclusive ion matrix (EIM) of *glycyrrhiza inflata* Bat. (GIB).

t_R_/min	m/z	I	t_R_/min	m/z	I	t_R_/min	m/z	I
25.30	270.32	3,62,901	32.39	719.28	7,101	16.94	200.38	3,428
24.02	675.26	17,204	23.18	527.21	6,301	30.56	727.32	2,827
23.30	865.36	17,077	32.65	761.33	5,254	29.21	425.20	2,749
29.16	761.33	13,591	19.17	353.14	4,823	11.50	355.12	2,601
24.89	707.28	10,898	25.66	205.09	4,686	27.28	298.35	2,329
18.58	623.22	10,573	24.22	707.28	4,448	25.32	270.61	2,215
18.82	987.49	7,350	25.40	355.15	3,979	—	—	—

#### 3.5.2 Identification of exclusive ion matrix

Based on the hierarchical matching strategy (HMS), further matching analysis was performed using the exclusive ion matrix (EIM) on the basis of the initial matching with the common ion matrix (CIM) (Wu et al., 2025). Table 9 shows the matching results of GUF analytical samples based on the EIMs of GUF and GIB.

**TABLE 9:** The MC and CIR results of 10 batches of GUF analytical samples.

Herbs	Lot	Match count	EIM of GUF/GIB	MC (%)	CIR
GUF	GUF01	20	GUF-20	100.0	+∞
		0	GIB-20	0	
	GUF05	20	GUF-20	100.0	+∞
		0	GIB-20	0	
	GUF07	18	GUF-20	90.0	+∞
		0	GIB-20	0	
	GUF08	19	GUF-20	95.0	+∞
		0	GIB-20	0	
	GUF11	19	GUF-20	95.0	+∞
		0	GIB-20	0	
	GUF12	18	GUF-20	90.0	+∞
		0	GIB-20	0	
	GUF16	16	GUF-20	80.0	+∞
		0	GIB-20	0	
	GUF20	18	GUF-20	90.0	+∞
		0	GIB-20	0	
	GUF25	18	GUF-20	90.0	+∞
		0	GIB-20	0	
	GUF30	16	GUF-20	80.0	+∞
		0	GIB-20	0	
	GUF31	17	GUF-20	85.0	+∞
		0	GIB-20	0	​
	GUF32	19	GUF-20	95.0	+∞
		0	GIB-20	0	
	GUF33	18	GUF-20	90.0	17.0
		1	GIB-20	5.0	
	GUF34	20	GUF-20	100.0	19.0
		1	GIB-20	5.0	
	GUF35	16	GUF-20	80.0	+∞
		0	GIB-20	0	

As presented in Table 9, the MC values of all GUF samples were no less than 80.0% when aligned with the EIM of GUF, whereas the MC values dropped to 5% when matched against the EIM of GIB. Meanwhile, the CIR values reflecting the change degree in MC were greater than 17.0, demonstrating a highly significant improvement in matching confidence and the EIMs of GUF and GIB exhibit stronger specificity and enable more accurate authentication of GUF. In addition, Table 10 shows the MC and CIR results of 10 batches of GIB analytical samples.

**TABLE 10:** The MC and CIR results of 10 batches of GIB analytical samples.

Herbs	Lot	Match count	EIM of GUF/GIB	MC (%)	CIR
GIB	GIB02	20	GIB-20	100.0	19.0
		1	GUF-20	5.0	
	GIB03	18	GIB-20	90.0	17.0
		1	GUF-20	5.0	
	GIB08	18	GIB-20	90.0	17.0
		1	GUF-20	5.0	
	GIB10	15	GIB-20	75.0	14.0
		1	GUF-20	5.0	
	GIB13	20	GIB-20	100.0	19.0
		1	GUF-20	5.0	
	GIB14	19	GIB-20	95.0	18.0
		1	GUF-20	5.0	
	GIB15	17	GIB-20	85.0	+∞
		0	GUF-20	0	
	GIB17	19	GIB-20	95.0	18.0
		1	GUF-20	5.0	
	GIB23	14	GIB-20	70.0	6.00
		2	GUF-20	10.0	
	GIB30	17	GIB-20	85.0	16.0
		1	GUF-20	5.0	
	GIB31	17	GIB-20	85.0	16.0
		1	GUF-20	5.0	
	GIB32	14	GIB-20	70.0	+∞
		0	GUF-20	0	
	GIB33	18	GIB-20	90.0	+∞
		0	GUF-20	0	
	GIB34	19	GIB-20	95.0	+∞
		0	GUF-20	0	
	GIB35	18	GIB-20	90.0	17.0
		1	GUF-20	5.0	

As illustrated in Table 10, the MCs of all GIB were no less than 70.0% when aligned with the EIM of GIB, whereas the MCs were no higher than 10.0% when matched against the EIM of GUF. Further, the MC values were categorized into two groups for non-parametric testing: Group X comprised the matching confidence of GUF and GIB against their own EIMs, while Group Y comprised the matching confidence of GUF and GIB against non-autologous EIMs. As shown in Supplementary Table S7, the *p*-value was less than 0.01, indicating a statistically significant difference. Meanwhile, the CIR values were no less than 6.00, indicating that the matching results based on the EIMs of GUF and GIB showed significant differences. It can realize more accurate authentication of GIB.

In summary, 15 batches of GUF and 15 batches of GIB were included as an external validation set. Following unified sample pretreatment and high resolution mass spectrometry acquisition, the MC and CIR were calculated for each sample. According to Tables 9 and 10, the minimum MC among all genuine samples was 70%. If the cutoff value were raised above 70% (e.g., to 80%), authentic samples with relatively low MC (such as GIB23) would be misclassified as non-target species, resulting in false negative outcomes (Wang X. R. et al., 2024). Conversely, if the cutoff were set below 70%, the risk of false positive identification would likewise increase. Accordingly, the preliminary candidate threshold for MC was established at 70% from the perspective of cutoff optimization. Regarding CIR values, the CIR values of genuine samples were generally much greater than 6.00, with only the borderline authentic sample GIB23 exhibiting a CIR value exactly equal to 6.00. Setting the CIR threshold above 6 would erroneously exclude some genuine samples, whereas a threshold below 6.00 might lead to false positive judgments. Therefore, the preliminary candidate cutoff for CIR was set at 6.00. Nevertheless, single indicator discrimination suffers from inherent limitations: neither MC alone nor CIR alone is sufficient for accurate identification. Consequently, a dual criterion combined discrimination strategy employing MC ≥ 70% and CIR ≥ 6.00 (logical AND) was adopted. Furthermore, receiver operating characteristic (ROC) analysis revealed that the dual indicator combined discrimination model with MC ≥ 70% combined with CIR ≥ 6.00 yielded an area under the curve (AUC) of 1.00, a maximum Youden’s index of 1.0, 100% sensitivity and 100% specificity, with no false negative or false positive results observed. Accordingly, 70% and 6.00 are the statistically optimal cutoff values for MC and CIR, respectively. This dual indicator combined model exhibits favorable discriminative performance and enables effective discrimination between GUF and GIB. Therefore, in the second stage of the HMS, it is required that the MC between the sample and its own EIM is no less than 70%, and the CIR is no less than 6.00, indicating a significant fold difference in MC values between the sample’s matching with its own EIM and that with non-self EIM. Overall, in accordance with the HMS, the matching analysis for the test samples is as follows: first, the matching analysis was performed based on the CIMs of GUF and GIB. The MC obtained from matching the test sample with the EIM of GUF (or GIB) was no less than 90% and higher than that from matching with the EIM of GIB (or GUF). Subsequently, when the matching was performed using EIM, the botanical origin of GRR test sample could be identified as GUF (or GIB) if the MC ≥ 70% and CIR ≥ 6.00 in its alignment with the EIM of GUF (or GIB).

#### 3.5.3 Market sample authentication

A total of five batches of GRR samples collected from the market were identified for botanical origins using CIM, EIM, and hierarchical matching strategy. Table 11 shows the matching results.

**TABLE 11:** The matching results of GRR market samples.

Herbs	Batches	CIM			EIM		
		Ions in GUF/GIB	MC (%)	CIR	Ions in GUF/GIB	MC (%)	CIR
GRR	GRR01	GUF-20	100	0.25	GUF-20	85	16.0
		GIB-20	80		GIB-20	5	
	GRR02	GUF-20	95	0.58	GUF-20	80	+∞
		GIB-20	60		GIB-20	0	
	GRR03	GUF-20	95	0.19	GUF-20	100	+∞
		GIB-20	80		GIB-20	0	
	GRR04	GUF-20	95	0.46	GUF-20	95	+∞
		GIB-20	65		GIB-20	0	
	GRR05	GUF-20	100	0.54	GUF-20	85	+∞
		GIB-20	65		GIB-20	0	
	GRR06	GUF-20	100	0.18	GUF-20	85	+∞
		GIB-20	85		GIB-20	0	
	GRR07	GUF-20	100	0.33	GUF-20	70	13.0
		GIB-20	75		GIB-20	5	
	GRR08	GUF-20	100	0.25	GUF-20	90	+∞
		GIB-20	80		GIB-20	0	
	GRR09	GUF-20	70	0.43	GUF-20	5	12.0
		GIB-20	100		GIB-20	60	
	GRR10	GUF-20	65	0.38	GUF-20	5	13.0
		GIB-20	90		GIB-20	70	

As shown in Table 11, expect for GRR09 and GRR10, the MCs of other GRR samples against the CIMs of GUF were higher than 90%, and were consistently higher than those against the CIMs of GIB. Furthermore, based on EIMs of GUF and GIB, the MCs of all GRRs against the EIMs of GUF were no less than 80% and the CIR ≥ 16.0. Therefore, according to the hierarchical matching strategy and identification criteria, the botanical origin of the commercial GRRs was determined to be GUF. At the same time, according to the hierarchical matching strategy (HMS), the GRR09 and GRR10 were identity as GIB.

#### 3.5.4 Exploration of differential components

Furthermore, the differential chemical constituents between GUF and GIB were explored by means of UPLC-QTOF-MS coupled with chemometric analysis (Yao et al., 2026). The chemometric analysis was conducted on 20 batches of GUF and 20 batches of GIB, with the results shown in Figure 3. As illustrated in Figure 3A, the GUF and GIB were located on opposite sides of the 95% confidence ellipse in the score plot, exhibiting obvious separation with R2X = 0.922 and Q2 = 0.876. These results demonstrated that there were inherent disparities in the chemical compositions of GUF and GIB (Ben Salem and Ben Abdelaziz, 2021). Figure 3B illustrates that GUF and GIB can be clearly distinguished using the OPLS-DA model, with evaluation parameters of R2X = 0.856, R2Y = 0.989, and Q2 = 0.992, confirming the favorable performance (Kang et al., 2022; Huang et al., 2018; Forsgren et al., 2025). Figure 3C indicates that the model is valid and not overfitted (Wang X. et al., 2024). Further, differential chemical markers were screened based on a VIP threshold of 10.0. The S-plots diagram in Figure 3D presented seven chemical markers with significant differences, in which four chemical markers for GIB and three chemical markers for GUF. Their corresponding VIP values in OPLS-DA model were 25.01, 21.15, 16.04 and 15.87 for the four GIB-derived markers, and 15.58, 13.21 and 11.71 for the three GUF-derived markers, all exceeding the set VIP threshold of 10.0. At the same time, the *p* value of CV-ANOVA is 3.6 × 10^−6^ (less than 0.01), indicating a statistically significant difference.

**FIGURE 3:**
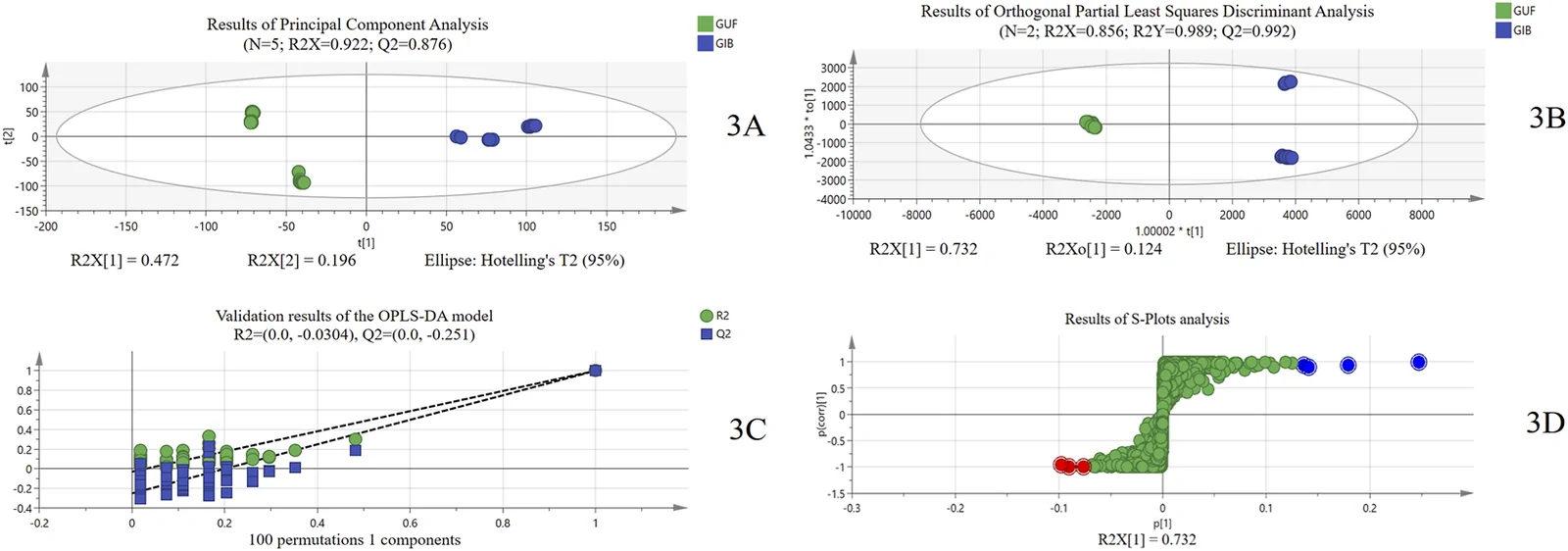
The results of chemometrics analysis of GUF and GIB (**(A)** shows PCA graph; **(B)** shows OPLS-DA results; **(C)** presents OPLS-DA model validation; **(D)** shows S-Plots results).

Through literature review and comparison with the self-established database, the four differential constituents (blue dots) of GIB were identified as Licochalcone A (23.23min_339.1590m/z, [M + H]^+^), Licochalcone B (11.93min_287.0914m/z, [M + H]^+^), Echinatin (14.67min_271.0915m/z, [M + H]^+^), Glabrol (26.88min_393.2059m/z, [M + H]^+^) and the three differential constituents (red dots) of GUF were identified as Licoricidin (27.92min_425.2303m/z, [M + H]^+^), Licoricone (26.01min_383.1491m/z [M + H]^+^) and Glycycoumarin (21.46min_369.1342m/z [M + H]^+^) (Chen et al., 2021; Kondo et al., 2007; Li et al., 2021; Li et al., 2016). Supplementary Figure S1 shows the ion distribution profiles of the four specific components in GIB and the results demonstrated that Licochalcone A, Licochalcone B, Echinatin, and Glabrol exhibited prominent mass spectrometric ion peaks in GIB, while they were nearly absent or present at extremely low levels in GUF samples. On the other hand, Supplementary Figure S2 shows the ion distribution profiles of the three specific components in GUF and the results demonstrated that Licoricidin, Licoricone and Glycycoumarin exhibited prominent mass spectrometric ion peaks in GUF, while they were nearly absent or present at extremely low levels in GIB samples. Above the differential components are highly consistent with results of the present study and are capable of providing additional support for the identification of GUF and GIB.

#### 3.5.5 Discussion

Market investigation indicated that, as medicinal and edible homologous Chinese medicinal herbs, GUF and GIB are the dominant original species of medicinal licorice circulating in the Chinese market (Nishida et al., 2025; Li et al., 2025). Given the practical application status of commercial licorice, this study preliminarily focuses on developing rapid identification methods for botanical origins of GUF and GIB, and innovatively constructing TCM-ID including CIM and EIM based on mass spectrometry. It aims to achieve precise differentiation between the two predominant licorice species for quality inspection and market supervision.

TCM-ID, which incorporated CIM and EIM, was proposed to identify the original species of licorice. In brief, TCM-ID is an identity marker for traditional Chinese medicines (TCMs) according to quantitative ion characterization of chemical components (He et al., 2025; Li et al., 2025; Wang et al., 2025). Essentially, TCM-ID is an ordered combination of digital chemical information based on high-resolution mass spectrometer (Liu et al., 2025; Zhang Q. et al., 2024; Liao et al., 2025). TCM-ID shifts the research focus from structural elucidation of individual compounds to the digital quantitative characterization of chemical constituents. This approach makes full use of massive data from numerous unidentified components, thereby breaking through the limitations of identification strategies relying only on single or a few marker compounds. On the other hand, compared with previous studies, the advantages of TCM-ID are reflected in CIM and EIM. The CIM fully takes into account the variations caused by multiple factors, such as cultivation years, geographical origins, and production batches, and screens stably and repeatedly detected ion features. Based on the CIM, the EIM further characterizes the differential ion information between GUF and GIB. Accurate species identification can be achieved only when all matching criteria of the two matrices are satisfied simultaneously. At the same time, the hierarchical matching strategy (HMS) was proposed to assist origin identification. With the CIM as the preliminary screening basis and followed by verification through the exclusive ion matrix EIM, hierarchical progressive judgment was realized to effectively reduce misjudgment caused by interfering factors. Furthermore, the combined criteria of stable universal ions and species-specific differential ions were adopted to avoid the limitations of single-index identification, thereby achieving accurate discrimination of GUF and GIB. As far as evaluation parameters are concerned, CIR is innovatively established on the basis of MC. MC means how close the test sample matches TCM-ID. CIR reflects the variation of matching credibility. In general, a higher CIR value indicates more significant origin differences. Through multi-batch sample testing, a cutoff value of CIR≥6.00 was established for EIM matching.

In the stages of sample pretreatment and mass spectrometry analysis, a comparative extraction evaluation was conducted using pure methanol, 75% methanol, 50% methanol and water. The results demonstrated that 75% methanol exhibited the optimal compatibility with high-resolution mass spectrometry, especially ESI conditions, with low baseline interference and the largest number of detectable ions. Compared with the ESI- mode, GUF and GIB presented more abundant ion information in the ESI + mode. Meanwhile, the ion responses at capillary voltages of 2.0 kV, 2.5 kV, 3.0 kV and 3.5 kV were investigated. The results indicated that the overall ion response reached the maximum at 3.0 kV. On the other hand, in the identification and matching process, the top 20 ions ranked by ion intensity and stably detected across multiple batches were uniformly screened and defined as CIM or EIM. In the identification analysis, the matching performance of the top 20, 30 and 50 ions was compared. The results demonstrated that adopting the top 20 ions ranked by ion intensity and stably presented in multiple batches as CIM and EIM yielded the optimal matching efficiency and accuracy.

The deviations in retention time (t_R_) and mass-to-charge ratio (m/z) are critical for the construction of CIM, EIM and subsequent matching identification. Excessively large deviations may cause false positive results, whereas overly strict deviation limits will lead to poor matching performance. Therefore, reasonable threshold values should be determined according to the actual sample injection conditions. In the multi-batch sample analysis, Licochalcone A (23.23 min, 339.1590 m/z, [M + H]^+^) and Licoricone (26.01 min, 383.1491 m/z, [M + H]^+^) were selected as reference characteristic ions. Throughout the analytical period, the tR deviation was less than 0.2 min, and the mass-to-charge ratio deviation was below 0.1 Da. Therefore, the acceptable deviation thresholds of tR and m/z were finally determined to be 0.2 min and 0.1 Da, respectively.

Further, based on traditional chemometric methods, the differential chemical constituents of GUF and GIB were explored to identify licorice botanical origins. The characteristic chemical components of GUF and GIB were finally identified. Licochalcone A (23.23min_339.1590m/z, [M + H]^+^), Licochalcone B (11.93min_287.0914m/z, [M + H]^+^), Echinatin (14.67min_271.0915m/z, [M + H]^+^) and Glabrol (26.88min_393.2059m/z, [M + H]^+^) are GIB’s characteristic chemical components. Licoricidin (27.92min_425.2303m/z, [M + H]^+^), Licoricone (26.01min_383.1491m/z, [M + H]^+^) and Glycycoumarin (21.46min_369.1342m/z [M + H]^+^) are GUF’s characteristic chemical components. The differential chemical components explored above are basically consistent with the findings reported in existing studies (Chen et al., 2021; Kondo et al., 2007; Li et al., 2021; Li et al., 2016; Wang et al., 2025). Based on the above differential chemical components, botanical origin identification of GUF and GIB can also be realized as an effective strategy. In further research, the combination of differential components, CIM and EIM will facilitate comprehensive identification from individual characteristic ions to overall chemical digital identity.

In this study, the identification of GUF and GIB were successfully realized based on TCM-ID (CIM and EIM) and HMS. Meanwhile, the characteristic chemical components of the two species were explored by means of chemometrics, which provides a reliable reference for the botanical origin identification of GUF and GIB. However, several limitations still exist in the present work. This study concentrated merely on GUF and GIB, with Glycyrrhiza glabra L. excluded from the experimental analysis. Therefore, the current research conclusions and relevant cutoff values in this study are only valid for the discrimination of GUF and GIB. Yet the methodological strategy based on TCM-ID (CIM and EIM) is highly replicable. Therefore, subsequent research will supplement Glycyrrhiza glabra specimens and multi-batch sample data, optimize CIM, EIM and relevant parameter thresholds, and ultimately achieve comprehensive and accurate identification of the three official licorice species.

## 4 Conclusion

In this paper, TCM-ID including CIM, EIM and hierarchical matching strategy (HMS) were innovatively proposed to identity glycyrrhiza species origins. Based on the identification Parameters-MC and CIR and HMS, the GUF and GIB were successfully identified and distinguished. Furthermore, among the market samples, 8 batches were authenticated as GUF and 2 batches as GIB. At the same time, chemometric difference analysis revealed that the characteristic components of GUF were Licoricidin, Licoricone and Glycycoumarin, while those of GIB were Licochalcone A, Licochalcone B, Echinatin and Glabrol. This study contributes to the botanical origin identification of GUF and GIB to a certain extent.

## Data Availability

The original contributions presented in the study are included in the article/Supplementary Material, further inquiries can be directed to the corresponding authors.
