# Supplementary material for: A novel digital identification strategy for licorice raw materials: TCM-ID with common/exclusive ion matrices and hierarchical matching
Source: Front Chem. 2026 Oct 1;14:1918761. doi: 10.3389/fchem.2026.1918761 (PMC13668877; doi:10.3389/fchem.2026.1918761)
Supplement: Supplementary file 1 [file DataSheet1.docx]

A Novel Digital Identification Strategy for Licorice Raw Materials: TCM-ID with Common/Exclusive Ion Matrices and Hierarchical Matching

Xianrui Wang, Yu Wu^†^, Lizhi Wan^†^, Jiating Zhang, Fangliang He, Wenguang Jing, Jia Chen^*^, Xianlong Cheng ^*^, Yongqiang Lin^*^

Institute for Control of Traditional Chinese Medicine and Ethnic Medicine, National Institutes for Food and Drug Control, Beijing, P. R. China 102629;

^†^Equal first-author contributions should be granted to the aforementioned authors.

*****Correspondence: chenjia@nifdc.org.cn; cxl@nifdc.org.cn; linyongqiang@nifdc.org.cn

Table S1 The detailed information of glycyrrhiza samples

| Glycyrrhiza samples | Batch | Usage | Origin | Years | Repository | Criteria |
| --- | --- | --- | --- | --- | --- | --- |
| *Glycyrrhiza uralensis* Fisch. | GUF01 | Analysis and verification (External validation) | Ningxia, China | 4 | NIFDC | Ch.P |
| *Glycyrrhiza uralensis* Fisch. | GUF02 | TCM-ID (EIM and CIM) construction (Training set) | Ningxia, China | 3 | NIFDC | Ch.P |
| *Glycyrrhiza uralensis* Fisch. | GUF03 | TCM-ID (EIM and CIM) construction (Training set) | Ningxia, China | 4 | NIFDC | Ch.P |
| *Glycyrrhiza uralensis* Fisch. | GUF04 | TCM-ID (EIM and CIM) construction (Training set) | Ningxia, China | 2 | NIFDC | Ch.P |
| *Glycyrrhiza uralensis* Fisch. | GUF05 | Analysis and verification (External validation) | Ningxia, China | 2 | NIFDC | Ch.P |
| *Glycyrrhiza uralensis* Fisch. | GUF06 | TCM-ID (EIM and CIM) construction (Training set) | Neimenggu, China | 4 | NIFDC | Ch.P |
| *Glycyrrhiza uralensis* Fisch. | GUF07 | Analysis and verification (External validation) | Neimenggu, China | 3 | NIFDC | Ch.P |
| *Glycyrrhiza uralensis* Fisch. | GUF08 | Analysis and verification (External validation) | Neimenggu, China | 2 | NIFDC | Ch.P |
| *Glycyrrhiza uralensis* Fisch. | GUF09 | TCM-ID (EIM and CIM) construction (Training set) | Neimenggu, China | 4 | NIFDC | Ch.P |
| *Glycyrrhiza uralensis* Fisch. | GUF10 | TCM-ID (EIM and CIM) construction (Training set) | Neimenggu, China | 2 | NIFDC | Ch.P |
| *Glycyrrhiza uralensis* Fisch. | GUF11 | Analysis and verification (External validation) | Gansu, China | 2 | NIFDC | Ch.P |
| *Glycyrrhiza uralensis* Fisch. | GUF12 | Analysis and verification (External validation) | Gansu, China | 1 | NIFDC | Ch.P |
| *Glycyrrhiza uralensis* Fisch. | GUF13 | TCM-ID (EIM and CIM) construction (Training set) | Xinjiang, China | 2 | NIFDC | Ch.P |
| *Glycyrrhiza uralensis* Fisch. | GUF14 | TCM-ID (EIM and CIM) construction (Training set) | Xinjiang, China | 3 | NIFDC | Ch.P |
| *Glycyrrhiza uralensis* Fisch. | GUF15 | TCM-ID (EIM and CIM) construction (Training set) | Xinjiang, China | 4 | NIFDC | Ch.P |
| *Glycyrrhiza uralensis* Fisch. | GUF16 | Analysis and verification (External validation) | Xinjiang, China | 2 | NIFDC | Ch.P |
| *Glycyrrhiza uralensis* Fisch. | GUF17 | TCM-ID (EIM and CIM) construction (Training set) | Xinjiang, China | 4 | NIFDC | Ch.P |
| *Glycyrrhiza uralensis* Fisch. | GUF18 | TCM-ID (EIM and CIM) construction (Training set) | Xinjiang, China | 4 | NIFDC | Ch.P |
| *Glycyrrhiza uralensis* Fisch. | GUF19 | TCM-ID (EIM and CIM) construction (Training set) | Shanxi, China | 5 | NIFDC | Ch.P |
| *Glycyrrhiza uralensis* Fisch. | GUF20 | Analysis and verification (External validation) | Shanxi, China | 3 | NIFDC | Ch.P |
| *Glycyrrhiza uralensis* Fisch. | GUF21 | TCM-ID (EIM and CIM) construction (Training set) | Shanxi, China | 2 | NIFDC | Ch.P |
| *Glycyrrhiza uralensis* Fisch. | GUF22 | TCM-ID (EIM and CIM) construction (Training set) | Shanxi, China | 4 | NIFDC | Ch.P |
| *Glycyrrhiza uralensis* Fisch. | GUF23 | TCM-ID (EIM and CIM) construction (Training set) | Shanxi, China | 2 | NIFDC | Ch.P |
| *Glycyrrhiza uralensis* Fisch. | GUF24 | TCM-ID (EIM and CIM) construction (Training set) | Shan'Xi, China | 2 | NIFDC | Ch.P |
| *Glycyrrhiza uralensis* Fisch. | GUF25 | Analysis and verification (External validation) | Liaoning, China | 4 | NIFDC | Ch.P |
| *Glycyrrhiza uralensis* Fisch. | GUF26 | TCM-ID (EIM and CIM) construction (Training set) | Liaoning, China | 5 | NIFDC | Ch.P |
| *Glycyrrhiza uralensis* Fisch. | GUF27 | TCM-ID (EIM and CIM) construction (Training set) | Liaoning, China | 3 | NIFDC | Ch.P |
| *Glycyrrhiza uralensis* Fisch. | GUF28 | TCM-ID (EIM and CIM) construction (Training set) | Liaoning, China | 2 | NIFDC | Ch.P |
| *Glycyrrhiza uralensis* Fisch. | GUF29 | TCM-ID (EIM and CIM) construction (Training set) | Liaoning, China | 4 | NIFDC | Ch.P |
| *Glycyrrhiza uralensis* Fisch. | GUF30 | Analysis and verification (External validation) | Liaoning, China | 2 | NIFDC | Ch.P |
| *Glycyrrhiza uralensis* Fisch. | GUF31 | Analysis and verification (External validation) | Shan'Xi, China | 4 | NIFDC | Ch.P |
| *Glycyrrhiza uralensis* Fisch. | GUF32 | Analysis and verification (External validation) | Ningxia, China | 3 | NIFDC | Ch.P |
| *Glycyrrhiza uralensis* Fisch. | GUF33 | Analysis and verification (External validation) | Neimenggu, China | 2 | NIFDC | Ch.P |
| *Glycyrrhiza uralensis* Fisch. | GUF34 | Analysis and verification (External validation) | Liaoning, China | 3 | NIFDC | Ch.P |
| *Glycyrrhiza uralensis* Fisch. | GUF35 | Analysis and verification (External validation) | Shanxi, China | 2 | NIFDC | Ch.P |
| *Glycyrrhiza inflata* Bat*.* | GIB01 | TCM-ID (EIM and CIM) construction (Training set) | Xinjiang, China | 2 | NIFDC | Ch.P |
| *Glycyrrhiza inflata* Bat*.* | GIB02 | Analysis and verification (External validation) | Xinjiang, China | 3 | NIFDC | Ch.P |
| *Glycyrrhiza inflata* Bat*.* | GIB03 | Analysis and verification (External validation) | Xinjiang, China | 4 | NIFDC | Ch.P |
| *Glycyrrhiza inflata* Bat*.* | GIB04 | TCM-ID (EIM and CIM) construction (Training set) | Xinjiang, China | 4 | NIFDC | Ch.P |
| *Glycyrrhiza inflata* Bat*.* | GIB05 | TCM-ID (EIM and CIM) construction (Training set) | Xinjiang, China | 2 | NIFDC | Ch.P |
| *Glycyrrhiza inflata* Bat*.* | GIB06 | TCM-ID (EIM and CIM) construction (Training set) | Xinjiang, China | 3 | NIFDC | Ch.P |
| *Glycyrrhiza inflata* Bat*.* | GIB07 | TCM-ID (EIM and CIM) construction (Training set) | Xinjiang, China | 2 | NIFDC | Ch.P |
| *Glycyrrhiza inflata* Bat*.* | GIB08 | Analysis and verification (External validation) | Xinjiang, China | 4 | NIFDC | Ch.P |
| *Glycyrrhiza inflata* Bat*.* | GIB09 | TCM-ID (EIM and CIM) construction (Training set) | Xinjiang, China | 2 | NIFDC | Ch.P |
| *Glycyrrhiza inflata* Bat*.* | GIB10 | Analysis and verification (External validation) | Xinjiang, China | 2 | NIFDC | Ch.P |
| *Glycyrrhiza inflata* Bat*.* | GIB11 | TCM-ID (EIM and CIM) construction (Training set) | Xinjiang, China | 2 | NIFDC | Ch.P |
| *Glycyrrhiza inflata* Bat*.* | GIB12 | TCM-ID (EIM and CIM) construction (Training set) | Gansu, China | 4 | NIFDC | Ch.P |
| *Glycyrrhiza inflata* Bat*.* | GIB13 | Analysis and verification (External validation) | Gansu, China | 4 | NIFDC | Ch.P |
| *Glycyrrhiza inflata* Bat*.* | GIB14 | Analysis and verification (External validation) | Gansu, China | 4 | NIFDC | Ch.P |
| *Glycyrrhiza inflata* Bat*.* | GIB15 | Analysis and verification (External validation) | Gansu, China | 3 | NIFDC | Ch.P |
| *Glycyrrhiza inflata* Bat*.* | GIB16 | TCM-ID (EIM and CIM) construction (Training set) | Gansu, China | 3 | NIFDC | Ch.P |
| *Glycyrrhiza inflata* Bat*.* | GIB17 | Analysis and verification (External validation) | Gansu, China | 2 | NIFDC | Ch.P |
| *Glycyrrhiza inflata* Bat*.* | GIB18 | TCM-ID (EIM and CIM) construction (Training set) | Gansu, China | 3 | NIFDC | Ch.P |
| *Glycyrrhiza inflata* Bat*.* | GIB19 | TCM-ID (EIM and CIM) construction (Training set) | Gansu, China | 3 | NIFDC | Ch.P |
| *Glycyrrhiza inflata* Bat*.* | GIB20 | TCM-ID (EIM and CIM) construction (Training set) | Gansu, China | 3 | NIFDC | Ch.P |
| *Glycyrrhiza inflata* Bat*.* | GIB21 | TCM-ID (EIM and CIM) construction (Training set) | Gansu, China | 4 | NIFDC | Ch.P |
| *Glycyrrhiza inflata* Bat*.* | GIB22 | TCM-ID (EIM and CIM) construction (Training set) | Gansu, China | 2 | NIFDC | Ch.P |
| *Glycyrrhiza inflata* Bat*.* | GIB23 | Analysis and verification (External validation) | Xinjiang, China | 3 | NIFDC | Ch.P |
| *Glycyrrhiza inflata* Bat*.* | GIB24 | TCM-ID (EIM and CIM) construction (Training set) | Xinjiang, China | 2 | NIFDC | Ch.P |
| *Glycyrrhiza inflata* Bat*.* | GIB25 | TCM-ID (EIM and CIM) construction (Training set) | Xinjiang, China | 4 | NIFDC | Ch.P |
| *Glycyrrhiza inflata* Bat*.* | GIB26 | TCM-ID (EIM and CIM) construction (Training set) | Xinjiang, China | 4 | NIFDC | Ch.P |
| *Glycyrrhiza inflata* Bat*.* | GIB27 | TCM-ID (EIM and CIM) construction (Training set) | Gansu, China | 4 | NIFDC | Ch.P |
| *Glycyrrhiza inflata* Bat*.* | GIB28 | TCM-ID (EIM and CIM) construction (Training set) | Gansu, China | 2 | NIFDC | Ch.P |
| *Glycyrrhiza inflata* Bat*.* | GIB29 | TCM-ID (EIM and CIM) construction (Training set) | Gansu, China | 3 | NIFDC | Ch.P |
| *Glycyrrhiza inflata* Bat*.* | GIB30 | Analysis and verification (External validation) | Gansu, China | 4 | NIFDC | Ch.P |
| *Glycyrrhiza inflata* Bat*.* | GIB31 | Analysis and verification (External validation) | Gansu, China | 3 | NIFDC | Ch.P |
| *Glycyrrhiza inflata* Bat*.* | GIB32 | Analysis and verification (External validation) | Gansu, China | 4 | NIFDC | Ch.P |
| *Glycyrrhiza inflata* Bat*.* | GIB33 | Analysis and verification (External validation) | Xinjiang, China | 2 | NIFDC | Ch.P |
| *Glycyrrhiza inflata* Bat*.* | GIB34 | Analysis and verification (External validation) | Gansu, China | 4 | NIFDC | Ch.P |
| *Glycyrrhiza inflata* Bat*.* | GIB35 | Analysis and verification (External validation) | Xinjiang, China | 4 | NIFDC | Ch.P |
| Commercial licorice | GRR01 | Identification analysis (Blind test) | — | — | NIFDC | — |
| Commercial licorice | GRR02 | Identification analysis (Blind test) | — | — | NIFDC | — |
| Commercial licorice | GRR03 | Identification analysis (Blind test) | — | — | NIFDC | — |
| Commercial licorice | GRR04 | Identification analysis (Blind test) | — | — | NIFDC | — |
| Commercial licorice | GRR05 | Identification analysis (Blind test) | — | — | NIFDC | — |
| Commercial licorice | GRR06 | Identification analysis (Blind test) | — | — | NIFDC | — |
| Commercial licorice | GRR07 | Identification analysis (Blind test) | — | — | NIFDC | — |
| Commercial licorice | GRR08 | Identification analysis (Blind test) | — | — | NIFDC | — |
| Commercial licorice | GRR09 | Identification analysis (Blind test) | — | — | NIFDC | — |
| Commercial licorice | GRR10 | Identification analysis (Blind test) | — | — | NIFDC | — |

Table S2 The results of of mass accuracy evaluation

| Compound | Molecular Formula | Adduct Ion | Calculated m/z | Observed m/z | Error (ppm) |
| --- | --- | --- | --- | --- | --- |
| Licochalcone A | C_21_H_22_O_4_ | [M+H]^+^ | 339.1596 | 339.1590 | -1.8 |
| Licochalcone B | C_16_H_14_O_5_ | [M+H]^+^ | 287.0919 | 287.0914 | -1.7 |
| Echinatin | C_16_H_14_O_4_ | [M+H]^+^ | 271.0970 | 271.0975 | 1.8 |
| Glycycoumarin | C_21_H_20_O_6_ | [M+H]^+^ | 369.1338 | 369.1342 | 1.1 |
| Leucine enkephalin | C_28_H_37_N_5_O_7_ | [M+H]^+^ | 556.2771 | 556.2782 | 2.0 |

Table S3 The results of of precision evaluation

| Number of injections | Point A | | Point B | | Point C | | Point D | |
| --- | --- | --- | --- | --- | --- | --- | --- | --- |
|  | t_R_ (min) | m/z | t_R_ (min) | m/z | t_R_ (min) | m/z | t_R_ (min) | m/z |
| 1 | 3.73 | 262.1907 | 15.58 | 985.4706 | 21.45 | 369.1347 | 31.91 | 439.2486 |
| 2 | 3.73 | 262.1908 | 15.59 | 985.4712 | 21.47 | 369.1352 | 31.92 | 439.2491 |
| 3 | 3.74 | 262.1910 | 15.61 | 985.4698 | 21.46 | 369.1349 | 31.91 | 439.2493 |
| 4 | 3.73 | 262.1909 | 15.59 | 985.4711 | 21.47 | 369.1351 | 31.93 | 439.2489 |
| 5 | 3.73 | 262.1908 | 15.59 | 985.4705 | 21.46 | 369.1361 | 31.95 | 439.2492 |
| 6 | 3.74 | 262.1915 | 15.59 | 985.4710 | 21.46 | 369.1345 | 31.93 | 439.2486 |
| Mean | 3.73 | 262.1910 | 15.59 | 985.4707 | 21.46 | 369.1351 | 31.93 | 439.2490 |
| Standard deviation | 0.005 | 0.000 | 0.010 | 0.001 | 0.008 | 0.001 | 0.015 | 0.000 |
| RSD (%) | 0.1383 | 0.0001 | 0.0631 | 0.0001 | 0.0351 | 0.0002 | 0.0475 | 0.0001 |

Table S4 The results of repeatability evaluation

| Replicate samples | Point E | | Point F | | Point G | | Point H | |
| --- | --- | --- | --- | --- | --- | --- | --- | --- |
|  | t_R_ (min) | m/z | t_R_ (min) | m/z | t_R_ (min) | m/z | t_R_ (min) | m/z |
| 1 | 8.35 | 257.0812 | 19.14 | 369.1349 | 25.32 | 317.1024 | 38.21 | 284.2957 |
| 2 | 8.33 | 257.0813 | 19.14 | 369.1348 | 25.33 | 317.1026 | 38.19 | 284.2953 |
| 3 | 8.36 | 257.0811 | 19.16 | 369.1350 | 25.34 | 317.1022 | 38.19 | 284.2929 |
| 4 | 8.33 | 257.0815 | 19.16 | 369.1349 | 25.30 | 317.1024 | 38.16 | 284.2946 |
| 5 | 8.34 | 257.0808 | 19.15 | 369.1352 | 25.32 | 317.1027 | 38.19 | 284.2935 |
| 6 | 8.34 | 257.0811 | 19.16 | 369.1346 | 25.34 | 317.1018 | 38.17 | 284.2943 |
| Mean | 8.34 | 257.0812 | 19.15 | 369.1349 | 25.33 | 317.1024 | 38.19 | 284.2944 |
| Standard deviation | 0.012 | 0.000 | 0.010 | 0.000 | 0.015 | 0.000 | 0.018 | 0.001 |
| RSD (%) | 0.1401 | 0.0001 | 0.0513 | 0.0001 | 0.0599 | 0.0001 | 0.0461 | 0.0004 |

Table S5 The results of stability evaluation

| Time (h) | Point I | | Point G | | Point K | | Point L | |
| --- | --- | --- | --- | --- | --- | --- | --- | --- |
|  | t_R_ (min) | m/z | t_R_ (min) | m/z | t_R_ (min) | m/z | t_R_ (min) | m/z |
| 0 h | 1.32 | 158.1172 | 10.20 | 273.0758 | 27.89 | 425.2331 | 32.16 | 421.1640 |
| 4 h | 1.33 | 158.1169 | 10.19 | 273.0757 | 27.91 | 425.2323 | 32.15 | 421.1647 |
| 8 h | 1.33 | 158.1165 | 10.21 | 273.0755 | 27.92 | 425.2303 | 32.17 | 421.1638 |
| 12 h | 1.34 | 158.1174 | 10.21 | 273.0756 | 27.92 | 425.2318 | 32.19 | 421.1644 |
| 24 h | 1.34 | 158.1165 | 10.23 | 273.0751 | 27.92 | 425.1326 | 32.20 | 421.1653 |
| 48 h | 1.32 | 158.1167 | 10.23 | 273.0760 | 27.93 | 425.1329 | 32.19 | 421.1643 |
| Mean | 1.33 | 158.1169 | 10.21 | 273.0756 | 27.92 | 425.1988 | 32.18 | 421.1644 |
| Standard deviation | 0.009 | 0.000 | 0.016 | 0.000 | 0.014 | 0.051 | 0.020 | 0.001 |
| RSD (%) | 0.6725 | 0.0002 | 0.1569 | 0.0001 | 0.0494 | 0.0120 | 0.0611 | 0.0001 |

Table S6 The results of the non-parametric test based on CIM

| Group | Median (P_25_，P_75_) | | Mann Whithey-U | Mann Whithey-Z | *p* |
| --- | --- | --- | --- | --- | --- |
|  | I (*n*=30) | II (*n*=30) |  |  |  |
| MC | 97.5 (90.0, 100.0) | 67.5 (58.8, 80.0) | 9.000 | -6.602 | 0.000* |

Table S7 The results of the non-parametric test based on EIM

| Group | Median (P_25_，P_75_) | | Mann Whithey-U | Mann Whithey-Z | *p* |
| --- | --- | --- | --- | --- | --- |
|  | X (*n*=30) | Y (*n*=30) |  |  |  |
| MC | 90.0 (85.0, 95.0) | 0.0 (0.0, 5.0) | 0.000 | -6.776 | 0.000* |

Fig.S1 The ion distribution profiles of Licochalcone A, Licochalcone B, Echinatin and Glabrol in *Glycyrrhiza uralensis* Fisch. (GUF) and *Glycyrrhiza inflata* Bat. (GIB)

Fig.S2 The ion distribution profiles of Licoricidin, Licoricone and Glycycoumarin in *Glycyrrhiza uralensis* Fisch. (GUF) and *Glycyrrhiza inflata* Bat. (GIB)
